# Site Investigations of the Lacustrine Clay in Taihu Lake, China, Using Self-Boring Pressuremeter Test

**DOI:** 10.3390/s21186026

**Published:** 2021-09-09

**Authors:** Bin Wang, Kang Liu, Yong Wang, Quan Jiang

**Affiliations:** 1 State Key Laboratory of Geomechanics and Geotechnical Engineering, Institute of Rock and Soil Mechanics, Chinese Academy of Sciences, Wuhan 430071, China; bwang@whrsm.ac.cn (B.W.); yongwang93@163.com (Y.W.); 2 University of Chinese Academy of Sciences, Beijing 100049, China; 3 College of Civil Engineering, Hefei University of Technology, Hefei 230009, China; k.liu@hfut.edu.cn

**Keywords:** plasticity index, self-boring pressuremeter test, shear modulus, shear strain, statistical analysis, undrained shear strength

## Abstract

Site investigations of the soils are considered very important for evaluation of the site conditions, as well as the design and construction for the project built in it. Taihu tunnel is thus far the longest tunnel constructed in the lake in China, with an entire length of over 10 km. However, due to the very insufficient site data obtained for the lacustrine clay in the Taihu lake area, a series of self-boring pressuremeter (SBPM) field tests was therefore carried out. Undrained shear strengths were deduced from the SBPM test, with the results showing generally higher than those obtained from the laboratory tests, which may be attributed to the disturbance to the soil mass during the sampling process. Degradation characteristics of the soil shear modulus (*G*_s_) were mainly investigated, via a thorough comparison between different soil layers, and generally, the shear modulus would cease its decreasing trends and become stable when the shear strain reaches over 1%. Meanwhile, it was found that a linear relationship between the plasticity index and the shear modulus, and between the decay rate of the shear modulus and the plasticity index as well, could be developed. Further statistical analysis over the undrained shear strength and shear modulus distribution of the soils shows that the undrained shear strength of the soils follows a normal distribution, while the shear modulus follows a log-normal distribution. More importantly, the spatial correlation length of the shear modulus is found much smaller than that of the undrained strength.

## 1. Introduction

Currently, investigations of soil properties are normally carried out by laboratory and in situ tests, with in situ tests considered more reliable [1,2,3,4], as they almost apply no disturbance to the soil. Self-boring pressuremeter (SBPM) is developed by Worth and Hughes in 1973 [5], and since then, it has been taken as one of the most important in situ testing techniques to investigate soil properties. During each test, both cavity expansion pressure and radial strain can be recorded, which renders an entire pressure-cavity–strain relationship curve, including both loading and unloading stages. In addition, the minimum measurement accuracy of radial displacement in SBPM can be as small as 1 μm [6,7], even higher than many laboratory tests, which therefore can provide mechanical parameters closer to the in situ states of the soils.

Based on the work of Palmer, Ladanyi, and Baguelin et al. [8,9,10], parameters, such as undrained shear strength, shear modulus, etc. can be deduced based on the SBPM test. Houlsby and Withers [11] analyzed soil properties from unloading and loading portions of pressuremeter curves and showed that the derived values of undrained shear strength and shear modulus are in good accuracy, compared to other tests’ results. Jefferies [12] proposed an approach to determine the horizontal geostatic stress of Beaufort Shelf clay from the SBPM test data. Bellotti et al. [13] proposed a method to correct the measured stiffness of SBPM in sand. Ferreira and Robertson [14,15] developed a method that incorporated nonlinear soil behavior, with both loading and unloading portions of the test to interpret SBPM results. Schnaid et al. [16] proposed a curve-fitting technique to interpret the granite saprolite parameters derived from the SBPM test, and the results were compared with other tests, including dilatometer and laboratory triaxial tests. By analyzing the test results obtained from SBPM in a sensitive Champlain clay of Quebec, Silvestri [17] concluded that the SBPM test did not overestimate the undrained shear strength, as opposed to the vane shear test results. Kayabasi [18] analyzed the limited pressure of pressuremeter test and the corrected SBP blows counts, respectively, where a new empirical equation between these two parameters has been developed. By using a power law of stiffness and strain, Whittle and Liu [19] developed a single equation to describe the strain and stress development of sand stiffness. Ahmadi and Keshmiri [20] suggested a new approach to interpret the in situ horizontal stress of SBPM via a finite difference model. The cavity pressure at a strain of 10% (*P*_10__%_) was selected to be the key parameters to derive in situ horizontal stress and new relationships were established between *P*_10__%_ and other soil parameters.

It should also be noted that natural soils are deemed heterogeneous. Christian and Baecher, Santoso et al., and Li et al. [21,22,23] have demonstrated the importance of considering soil heterogeneity in geotechnical engineering features, such as seepage, settlement, and slope stability. Firouzianbandpey et al. [24] investigated both the vertical and horizontal correlation lengths of sand layer deposits based on CPTu data from two different sites in the north of Denmark. De Gast et al. [25] analyzed the CPT data from a rural dyke in the Netherlands and assessed the vertical scale of fluctuation of the soils.

Therefore, two aspects were mainly focused on this paper—for one, a considerable amount of SBPM tests were carried out in Taihu Lake, China, aiming to obtain a straight-forward understanding of the soil parameters variation at the Taihu lake bed with respect to the depth, as well as the loading strain, etc.; for the other, by combining the statistical analysis, the test data were further interpreted from a probabilistic view, which can provide the designer necessary information to assess the safety condition of the geo-structures via probabilistic methods.

## 2. SBPM Test Apparatus and Test Site

**Figure 1:**
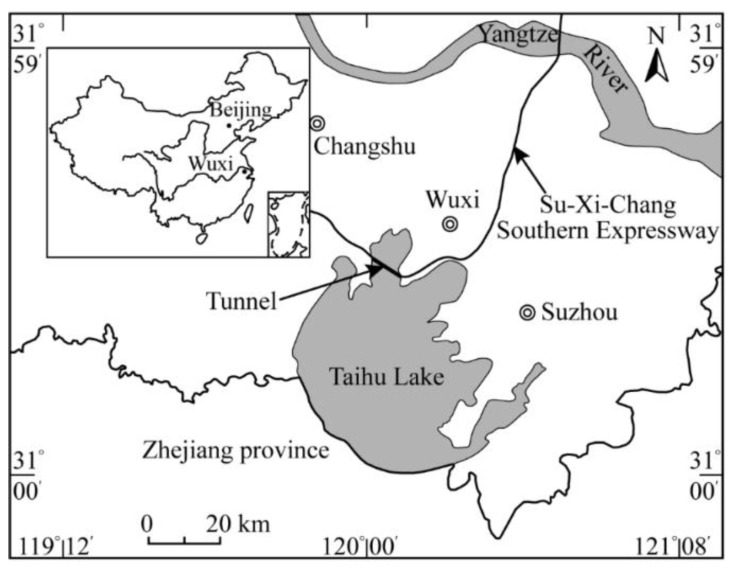
Location of the SBPM test site.

The test site is located in the Taihu lake area in Wuxi city, China, as shown in Figure 1, where the Su-Xi-Chang South highway crosses. This part of the highway is constructed as a tunnel by using the open-cut method, which is indicated by the bold line in Figure 1. It is so far the longest tunnel constructed in the lake in China, with a sub-water length of 10.67 km. In view of the variable mechanical properties of the lacustrine clay, more detailed parameters are needed by the ownership and designers for consideration of the construction safety. For this reason, the SBPM tests were conducted in the tunnel construction site of Taihu lake bed by Wuhan Institute of Rock and Soil Mechanics, Chinese Academy of Sciences.

**Figure 2:**
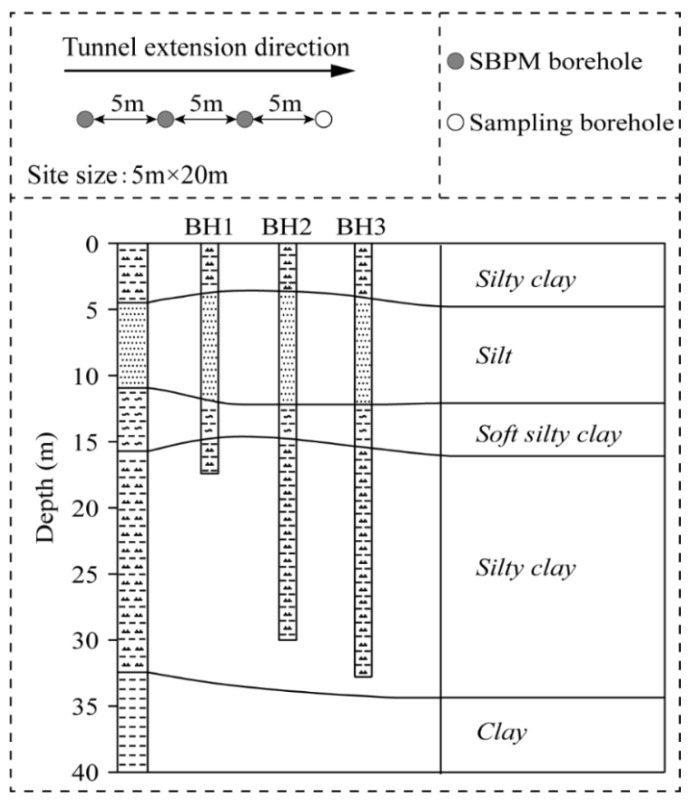
Borehole alignment and soil profile of the test site (Note: BH—SBPM borehole.).

The SBPM tests were conducted by using the Cambridge self-boring pressuremeter, which can estimate soil properties such as shear modulus, undrained shear strength, and horizontal stress, etc. Three sets of SBPM tests were carried out in the site, with a horizontal distance of 5 m between each borehole aligning in a straight line, as indicated by the solid circles shown in Figure 2. A sampling borehole, as represented by the hollow circle, was drilled as well for obtaining a rough picture of the prior information of the soil stratum. Tests were conducted, and the results were read once per meter, with an average depth of the three boreholes around 25 m, and the top elevation of each borehole was the same. The soil profile in this site resulted from the local geological maps, which consisted of the following: (i) silty clay in the depth interval of 0–4.5 m and 17–34 m, respectively; (ii) 4.5–12 m of silt; (iii) a 12–17 m of soft silty clay; (iv) 34–40 m of clay. Table 1 shows some basic physical properties of the soils in the test site.

## 3. Methods to Analyze the Test Results

### 3.1. Shear Modulus

Concerning the derivation of the shear modulus, there are two different methods, which are linear analysis and nonlinear analysis. In the linear analysis, the shear modulus from the unloading–reloading cycle, namely *G*_ur_, is a constant value, whereas the secant shear modulus *G*_s_ in the nonlinear analysis changes with the shear strain.

**Figure 3:**
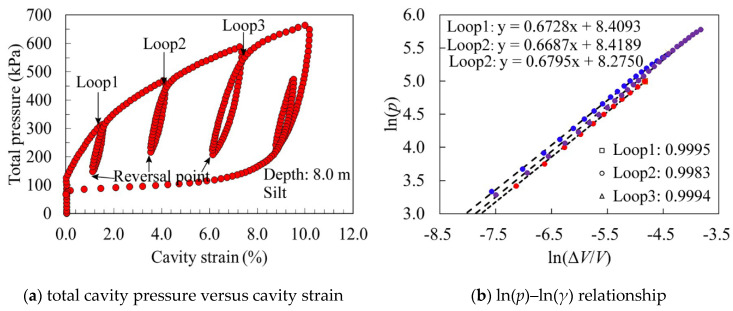
A representative SBPM test result curve.

By assuming the soil is perfectly elastic during the unloading process, the gradient of the unloading–reloading cycle is thereby twice the linear shear modulus (i.e., 2 *G*_ur_), where *G*_ur_ is also referred to as the unloading–reloading shear modulus [7,16]. Bolton and Whittle [26] showed that the power–law function can well describe the variation of the shear stress with strain, i.e.,

(1) $$\tau =\alpha \gamma^{\beta }$$

where α and β are the stiffness constant and elastic exponent, respectively, which can be derived from the reloading data of the unloading–reloading cycles. Three unloading–reloading cycles have been obtained in each SBPM test during the expansion phase, as shown in Figure 3a. In logarithmic coordinates, there is a strong linear correlation of total cavity pressure with the shear strain for reloading data (see Figure 3b). Therefore, the nonlinear deduction of secant shear modulus can be expressed as

(2) $$G_{s}=\frac{\tau }{\gamma }=\alpha \gamma^{\beta -1}$$

### 3.2. Undrained Shear Strength

The pressuremeter undrained shear strength, $c_{u}$, can be derived from the method proposed by Gibson and Anderson [27], where a relationship between the total pressure and the initial horizontal stress and undrained shear strength is established, as shown below.

(3) $$p=p_{0}+c_{u}[1+\ln(G/c_{u})]+c_{u}\ln(\Delta V/V)$$

in which p is the total pressure, $p_{0}$ is the initial horizontal stress, $c_{u}$ is the undrained shear strength, *G* is the shear modulus, and ΔV/V refers to the shear strain of the soil. Based on this equation, the relationship between the total pressure and the undrained shear strength can be plotted in a semi-logarithmic coordinate system. It should be noted that the well-known Gibson and Anderson solution is indeed for the undrained expansion of a cylindrical cavity in an elastic-perfectly plastic soil. However, a common practice is to assume that all the SBPM tests were carried out under the undrained conditions for clay. Meanwhile, assumptions of linear/nonlinear elastic behavior of the soil may lead to different solutions of the stresses and strains, but the result undrained shear strength, which equals to the ratio of the incremental total pressure over the incremental logarithmic shear strain of the soil, will not be affected, as the later part of the curve corresponds to the plastic phase and the slope of the curve is the undrained shear strength.

## 4. SBPM Test Results

The field test results of SBPM are interpreted in this section, in which the in situ horizontal stress, undrained shear strength, and shear modulus of the soils along the depth are analyzed. In particular, special focus is placed on the degradation of the shear modulus with shear strain. Moreover, by adopting probabilistic theories, the statistical characteristics and scale of fluctuations of the soils in the vertical direction are presented.

### 4.1. Stress–Strain Response of Soils

**Figure 4:**
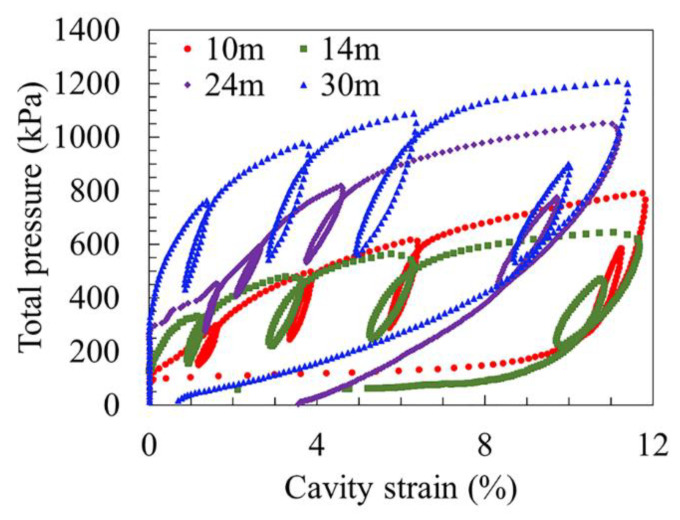
An illustrative diagram showing the relationship between the total pressure and the cavity strain.

For illustrations, the relationship between the shear stress and the shear strain obtained by using the SBPM at different depths is provided, as shown in Figure 4. It should be noted that at the very beginning, the curve rises along the vertical axis. The reason is that the deformation would not be shown until the expansion pressure *p* reaches the horizontal stress $\sigma_{h0}$. When the expansion pressure increases to the lift-off pressure *p*_0_, the soil can thereby deform and the expansion pressure increases with the cavity deformation. It can be clearly seen that the shear stress keeps increasing with the shear strain. As the depth increases, the curves generally distribute more upwards, showing a pattern of the shear stress positively related to the depth. It is also worth noting that the curve for the soils at the depth of 15.0 m is the flattest, rendering the smallest shear stress, compared to the rest samples. This may indicate a weak layer there, which can be verified by the geological map, as shown in Figure 2, where the soft silty clay is seen distributed from 12 to 17 m.

**Figure 5:**
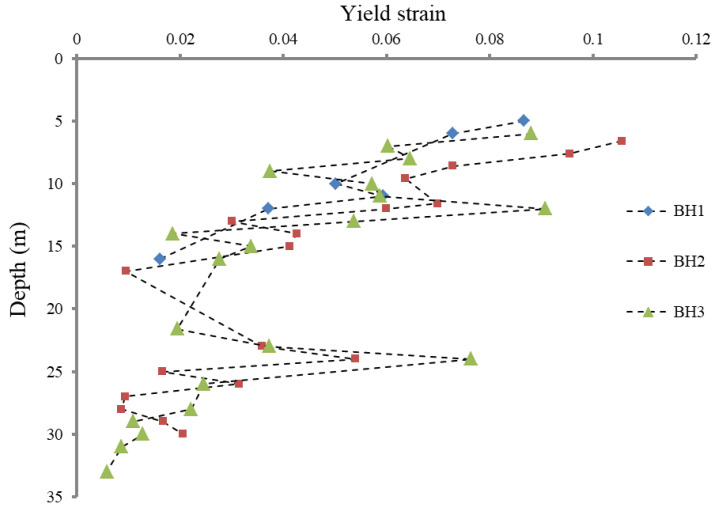
Yield strain distribution for different soils along the depth.

The yield strain distribution for different soils at different depths is shown in Figure 5, where the yield strain is defined as

(4) $$\gamma_{y}=\exp{\left[\left(\frac{p_{\mathrm{Limit}}-p_{0}}{c_{u}}\right)-\left(\frac{1}{\beta }\right)\right]}^{-1}$$

where $\gamma_{y}$ is the yield strain, which defines the boundary of the elastic behavior of the soil, *p*_0_ is the cavity reference pressure, and *p*_Limit_ is the limit pressure.

From the figure, a general decreasing tendency of the yield strain along the depth can be seen. As the depth increases, the soil is shown to be more quickly entered the plastic stage during the loading process. Another fact that can be concluded is that the soft silty clay has the smallest yield strain as a whole, while the silt possesses the largest yield strain. This may be due to higher structural integrity for the soft silty clay, thereby being more easily broken up to cause plastic deformation. It can also be noted that the yield strain is far larger than the results obtained by the triaxial tests, which represents a stress state of a single point in space. This may be due to the fact that the yield describes the stress state of an area of soils around the cavity, depending on the failure degree of the soil’s structural integrity.

### 4.2. Undrained Shear Strength

**Figure 6:**
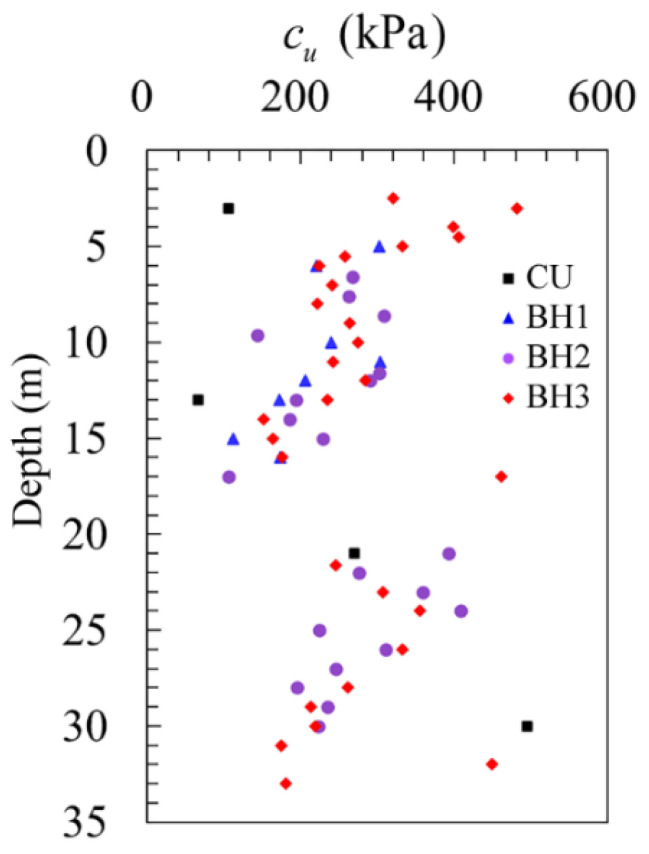
Undrained shear strength.

As described in Section 3.2, the pressuremeter undrained shear strength can be derived from the method proposed by Gibson and Anderson [27]. The variation of the undrained shear strength, *c_u_*, with depth is provided in Figure 6, and for comparison, a series of laboratory tests were conducted by using consolidated undrained (CU) triaxial tests, with the results being denoted by the square black dots. By comparison, the laboratory test results are generally lower than the in situ results. This may be due to the fact that the soil disturbance and stress relief during the sampling process. However, as a whole, the consistency between the in situ results and the laboratory results can, to some extent, prove the validity of the in situ measurement results.

**Figure 7:**
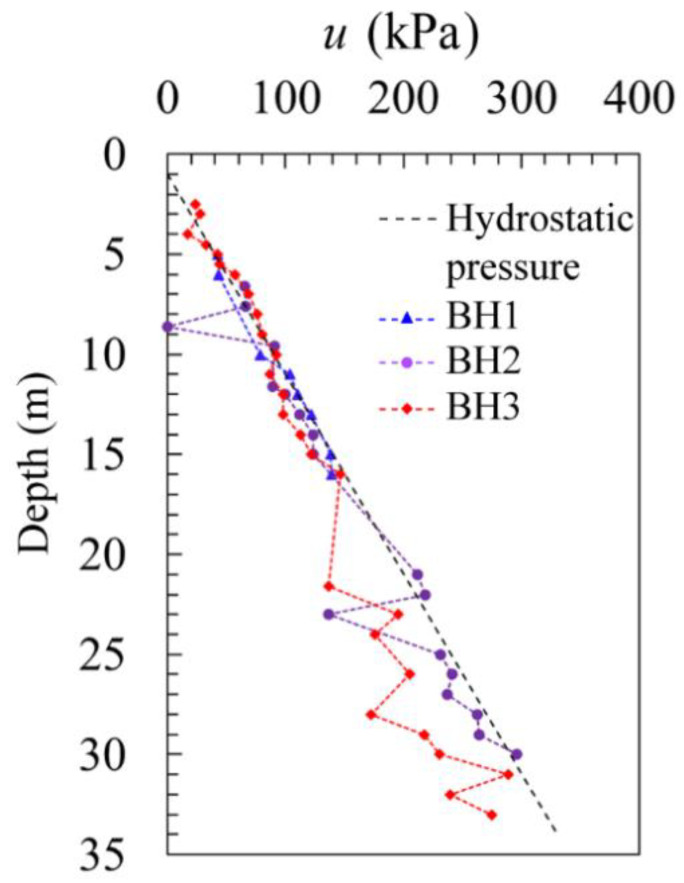
Pore water pressure.

It is a common practice to assume that all the SBPM tests were carried out under undrained conditions for clay for two reasons. For one, the clay normally has a very low permeability; for the other, the loading rate is relatively high, which may not enable the soil to have enough time to dissipate the pore pressure during the loading process. In this study, the pore water pressure *u* was also measured on the cavity wall, as illustrated in Figure 7, where the variation of pore water pressure in different soil layers along the depth is shown. A general consistent result with the hydrostatic pressure is shown, where the underground water table on the site is found roughly 1 m below the ground.

Therefore, to conclude, via the comparison with the laboratory test results, as well as the analytical solutions for the hydrostatic pressure distributions, the accuracy of the SBPM test can be well demonstrated.

### 4.3. In Situ Horizontal Stress

**Figure 8:**
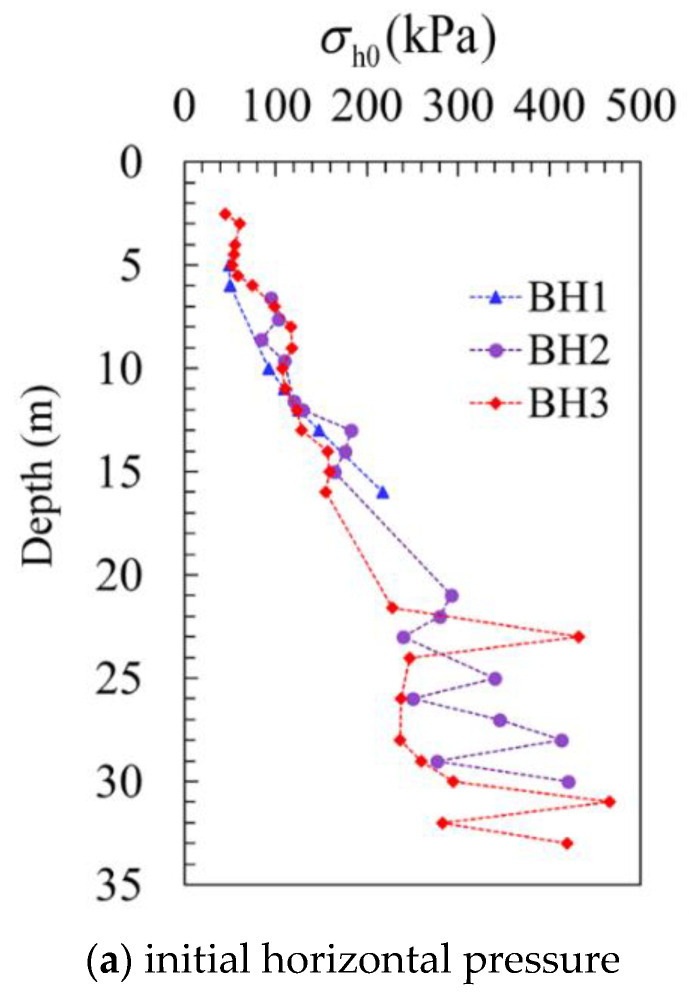

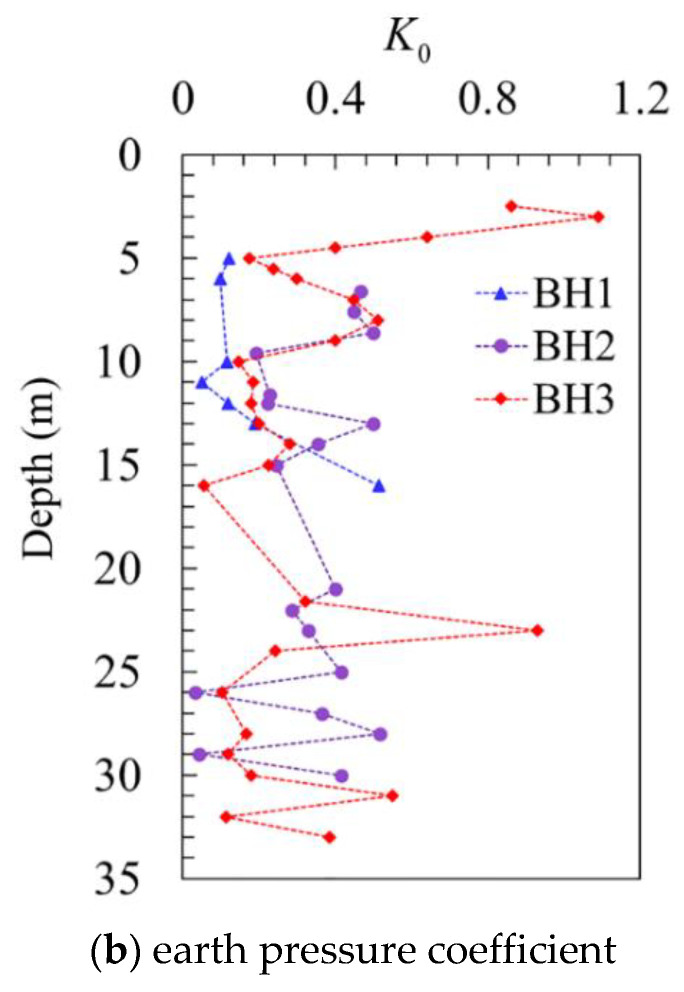
Initial horizontal stress distribution along the depth.

One of the main functions of the SBPM test is to measure the in situ horizontal stress. Assuming that there is no disturbance during the insertion of the SBPM probe when the probe reaches a certain depth, the pressure acting on the membrane of the probe is equal to the in situ horizontal pressure $\sigma_{h0}$. Figure 8a presents the result of in situ horizontal pressure, and it seems that $\sigma_{h0}$ increases with depth. It is worth noting that there is no significant difference between the three boreholes when the depth is less than 17 m, but obvious discrepancies can be seen when the depth is greater than 17 m.

By using the horizontal effective stress $\sigma_{h0}^{\prime }$ that can be calculated from the value of $\sigma_{h0}$, shown in Figure 8, and the vertical effective stress $\sigma_{v0}^{\prime }$, the coefficient of earth pressure at rest *K*_0_ can be derived as $K_{0}=\sigma_{h0}^{\prime }/\sigma_{v0}^{\prime }$. As depicted in Figure 8b, *K*_0_ is distributed within the range of 0.03 to 1.21, where for the measuring points near the ground surface of borehole 3, extremely large values are shown, which could be due to the traffic loads of the construction vehicles nearby. In general, the results of the three boreholes are pretty close when the depth is less than 17 m, while, in comparison, the difference of *K*_0_ is relatively larger when the depth is below 17 m.

### 4.4. Shear Modulus

#### 4.4.1. Degradation Characteristics of the Shear Modulus

**Figure 9:**
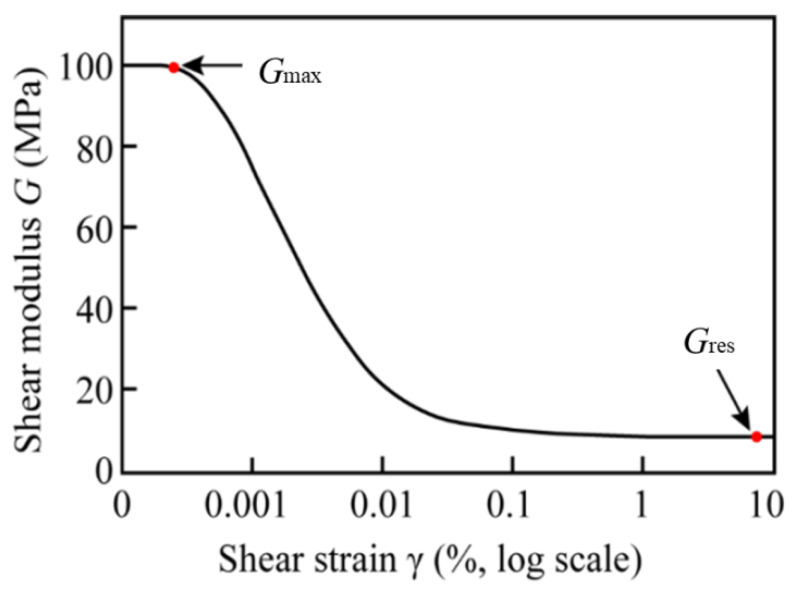
Schematic diagram of pressuremeter stiffness and shear strain (modified according to Whittle and Liu, 2013 [19]).

A schematic diagram of the stiffness change with strain is provided in Figure 9. It can be seen that the shear modulus is normally considered as a constant when the shear strain order is very small, and while the shear strain increases, the shear modulus decays rapidly, and finally reaches a constant. Therefore, for quantitative analysis of the shear modulus variation along the depth at different stages, the maximum shear modulus *G*_max_, and residual shear modulus *G*_res_, which corresponds to the smallest and largest shear strain measured in the SBPM tests for each measuring point, respectively, are assembled and plotted, as shown in Figure 9. For comparison, the unloading–reloading modulus *G*_ur_ is also included.

**Figure 10:**
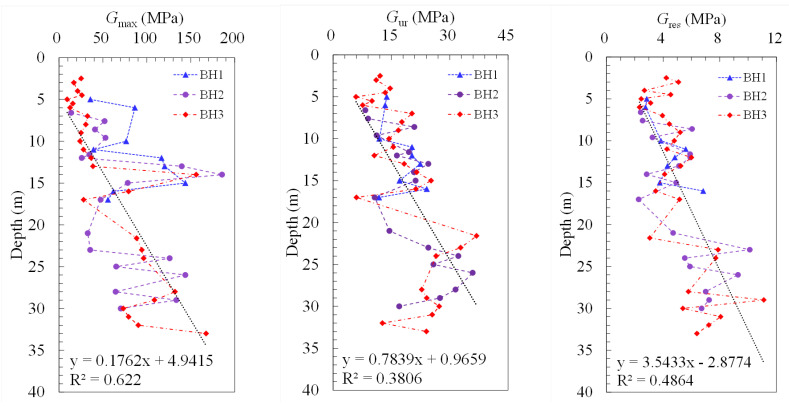
Distribution of the shear modulus along the depth.

A generally increasing trend of the shear modulus along the depth is shown in Figure 10. It is seen from the figures that, *G*_max_ ranges from 9.3 to 185.6 MPa, and *G*_res_ varies from 2.8 to 12.0 MPa, while *G*_ur_ is distributed from 6.0 to 46.9 MPa, which is in between. It may also be inferred from the figure that the heterogeneity of the soils increases along the depth in the Taihu lake clay, due to the fact that as the depth increases, the differences of the soil responses tested from the boreholes are seen to increase.

**Figure 11:**
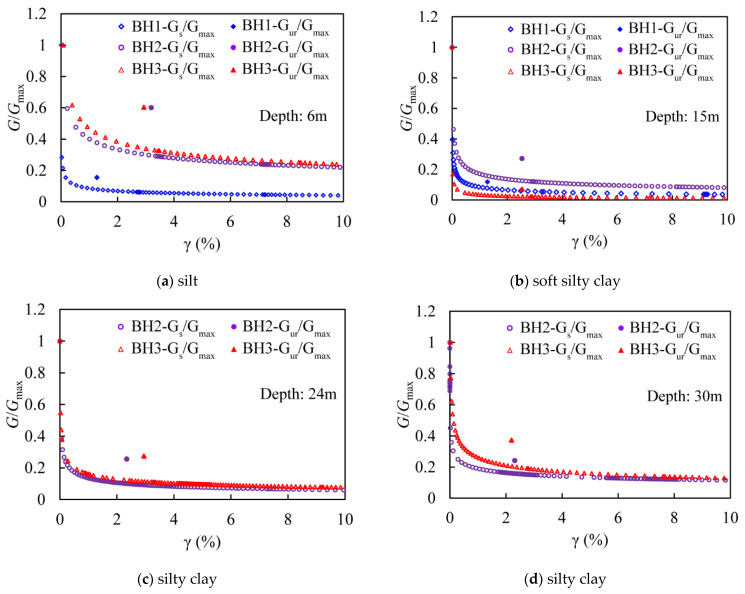
Normalized shear modulus of different soil types and depths.

More specifically, to explore the details of the degradation characteristics of each type of soil, four typical examples are selected and presented. In Figure 11, the relationship between the normalized shear modulus *G*_s_/*G*_max_ and the shear strain is shown, where the normalized unloading–reloading shear modulus *G*_ur_/*G*_max_ is also included. It can be seen that firstly, the shear modulus decreases with the increasing shear strain, and generally, a stable state can be attained when the shear strain γ > 1%. Moreover, the normalized unloading–reloading shear modulus *G*_ur_/*G*_max_ is generally distributed within the strain range of 1.0–3.2%. In contrast to the nonlinear modulus corresponding to the same strains, the linear ones are all shown to be located above the curves, and the differences among them gradually decrease as the strain increases. This may imply an overestimation of the structural safety when the shear strain is relatively small with direct use of *G*_ur_.

In addition, by comparing the sub-figures, it can also be found that the sharpest reduction of the shear modulus is seen in soft silty clay, where it can be seen that the modulus decreases rapidly within a very small range of strain increase, and moreover, the residual modulus is seen to be the lowest, compared to the other soil types as well. By summarizing the residual ones for different types of soils, it is found that the values vary from 0.01 to 0.24; for silt, the converged value ranges from 0.04 to 0.24, while this value varies from 0.01 to 0.08 for soft silty clay. The converged values of silty clay fall within the range of 0.08 to 0.23. Compared with silt and silty clay, the converged value of shear modulus in soft silty clay is obviously in a smaller range, which may indicate a relatively stronger structural property for soft silty clay. As for the discrepancies of the curves shown in Figure 11a, it may be due to quite a lot of shell coins accumulated in this layer, as seen on the site.

#### 4.4.2. Strain Influence on Distribution of Secant Shear Moduli

**Figure 12:**
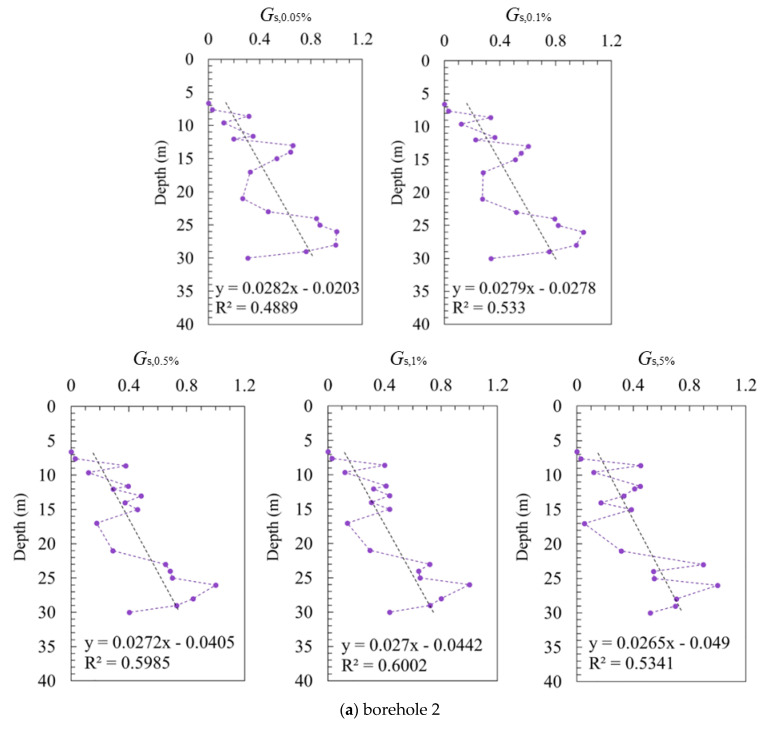

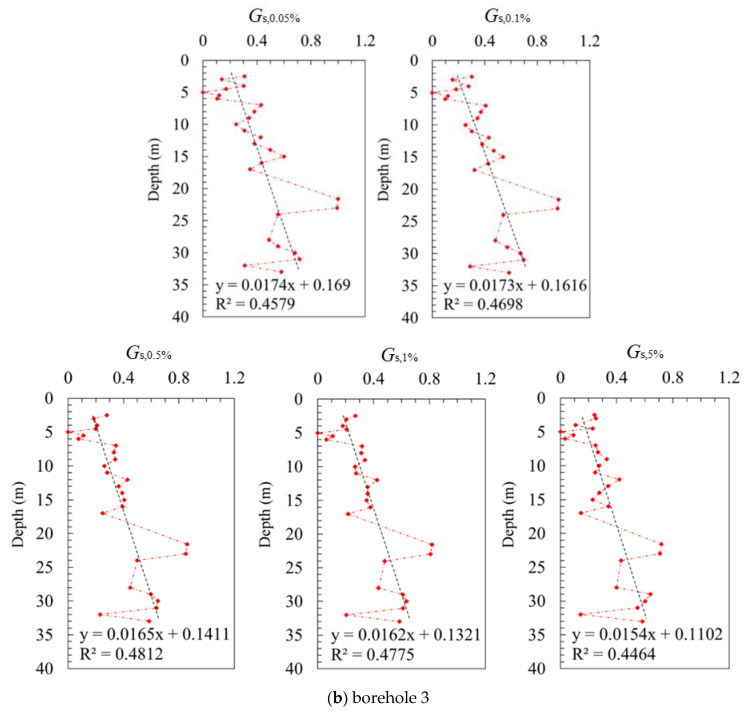
Distribution of normalized shear modulus ${\bar{G}}_{s}$ at different shear strains along the depth for boreholes 2 and 3.

As stated before, the secant modulus varies with the shear strain; therefore, to estimate the influence of the shear strain on the shear modulus, the secant shear modulus (*G*_s_) at different shear strains, i.e., γ = 0.05%, γ = 0.1%, γ = 0.5%, γ = 1%, and γ = 5%, are chosen and plotted against the depth, as shown in Figure 12. To study the increasing tendency of the shear modulus along the depth at different strains, the secant shear modulus is further normalized with respect to the depth,

(5) $${\bar{G}}_{s,n}=\frac{G_{s,n}-G_{\mathrm{res},n}}{G_{\max,n}-G_{\mathrm{res},n}}$$

where *G*_s_ is the measured shear modulus at a certain depth, and the subscript *n* corresponds to the strain when the shear modulus is measured, while *G*_max,*n*_ and *G*_res*,n*_ are the maximum and minimum measured shear modulus of all the depths at the shear strain *n*, respectively.

Figure 12 shows the variation of the normalized secant shear moduli (${\bar{G}}_{s}$) with the depth at different shear strains. It should be noted that due to the insufficient data obtained from borehole 1; therefore, only the data from boreholes 2 and 3 are summarized, and plotted separately to exclude the influence from borehole 1. Although a weak correlation of the modules for different depths is shown, which is mainly due to few single points deviated far away, a generally increasing tendency of the shear modulus with respect to the depth can still be seen at all the strains. This can also be attributed to the discontinuous data obtained by SBPM, as tests have been conducted per meter, but the density is already very high. A combination of other field tests, such as CPTU, can be incorporated, which may help improve the assessment of the shear modulus distribution.

A series of trend lines of ${\bar{G}}_{s}$ are constructed on basis of the scattered data. Although there is a little discrepancy between borehole 2 and borehole 3, what they have in common is that the slope of the trend line decreases with the increasing shear strain. As the strain increases from 0.05% to 5%, the slope of the trend lines for borehole 2 decreases from 0.0282 to 0.0265, and from 0.0174 to 0.0154 for borehole 3, respectively (see Figure 12a,b). This indicates that when the shear strain increases, the degree of the increasing tendency of the shear modulus slows down along the depth. This may be due to the degradation characteristics of the shear modulus with strain. When the shear strain is small, the discrepancies of the shear modulus in the depth direction are significant. With the increase in the shear strain, the difference gradually reduces. As a result, the slope of the trend line ${\bar{G}}_{s}$ shows a decreasing tendency with the increase in the shear strain.

#### 4.4.3. Relationship between Secant Shear Moduli and Plastic Index

**Figure 13:**
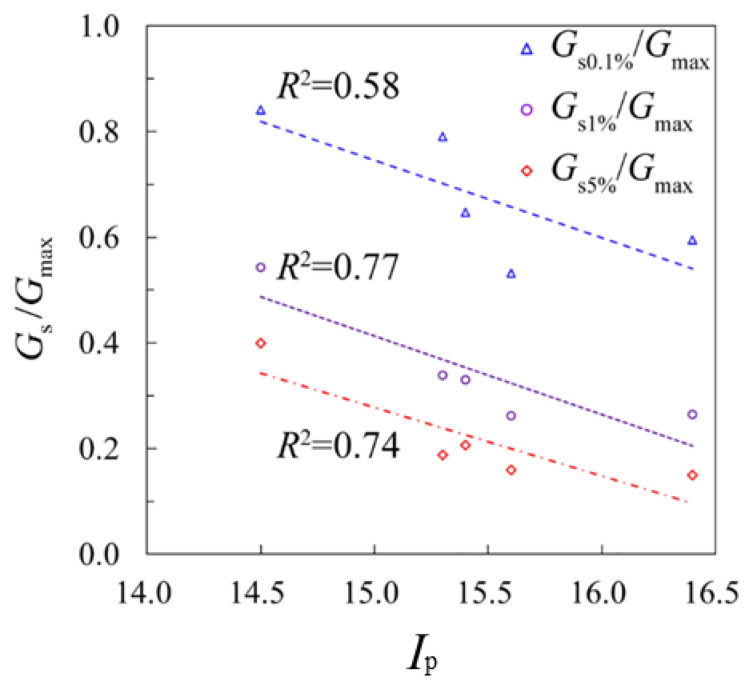
Correlation of *G*_s_/*G*_max_ with *I*_p._

The strength parameters are widely acknowledged to be closely related to the soil’s physical parameters [28,29,30,31]. In this paper, a relationship between the shear modulus and the plasticity index, i.e., *I*_p_, has been established. Laboratory tests of the plasticity index of different soil types have been performed, as presented in Table 1. The correlation of *G*_s_/*G*_max_ with plasticity index *I*_p_ under different shear strains is plotted in Figure 13, where generally a negative relationship is shown. As the plasticity index increases, *G*_s_/*G*_max_ decreases correspondingly, which means that by applying the same loaded strains, the higher the plasticity index is, the lower the shear modulus would be reached, i.e., a higher decay rate it is. Therefore, a simple conclusion can be made that the larger the plasticity index is, the higher the decay rate of the soil is.

**Figure 14:**
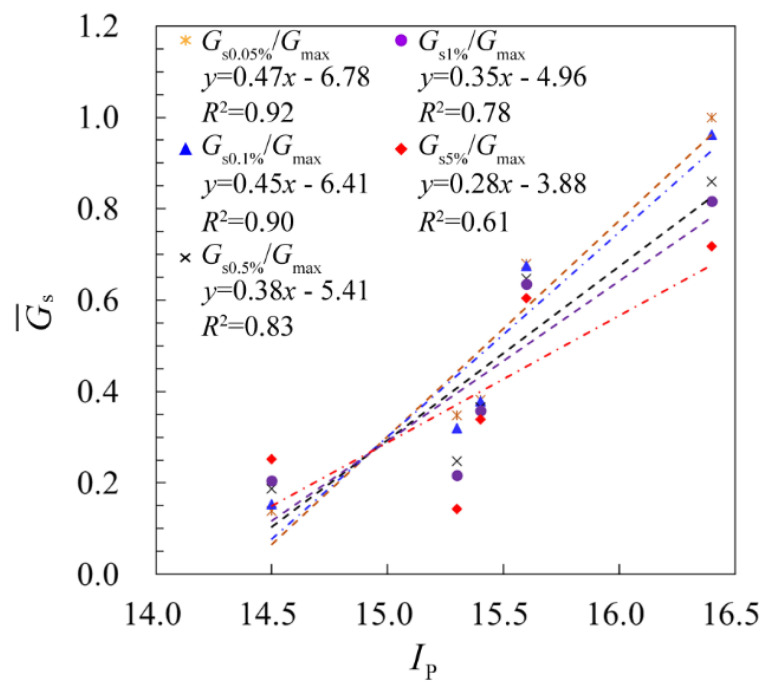
Correlation of normalized shear modulus with plasticity index *I_P_* at different shear strains (i.e., γ = 0.05%, γ = 0.1%, γ = 0.5%, γ = 1% and γ = 5%).

Figure 14 shows the relationship between ${\bar{G}}_{s}$ and *I*_p_ at different shear strains, where ${\bar{G}}_{s}$ is the normalized shear modulus with respect to the depth, as defined in Equation (4). It can be seen from the figure that generally a linearly positive relationship between ${\bar{G}}_{s}$ and *I*_p_ is presented in which, as the shear strain increases from 0.05% to 5%, the slope of the fitting curves decreases correspondingly, giving a slope of 0.472, 0.447, 0.380, 0.350 and 0.278, respectively. Two findings may be deduced from the figure. Firstly, as the plasticity index *I*_p_ increases, the normalized shear modulus ${\bar{G}}_{s}$, which is positively related to the secant shear modulus *G*_s_, increases as well, thereby indicating a positive relationship between the *I*_p_ and *G*_s_. In other words, the larger the plasticity index *I*_p_ is, the higher the shear modulus is. Secondly, with the increase in the shear strain, the increase rate of the shear modulus with respect to the plasticity index is gradually reduced, which means that the influence of the plasticity index on the shear modulus increase is reduced as the strain increases. Therefore, the shear modulus decay can be counted as a combination of the magnitude of the shear deformations and the change in the plasticity index, while obtaining a quantitative judge on which factor is more important can be a future topic.

### 4.5. Statistics Analysis of Secant Shear Modulus and Undrained Shear Strength

From the above-analyzed results, the statistics of the shear modulus and undrained shear strength can be estimated. Table 2 provides the mean, standard deviation, and coefficient of variation (COV) of the shear modulus at different shear strains and undrained shear strengths, where the COV denotes the degree of variation in soil properties across the test site. The higher COV implies the variability of the soil is more. It can be seen from the table that the coefficient of variation of the values is around 30%, which is in the range of 0.1–0.5, as normally reported in the literature [32,33].

An interesting finding is that the statistical properties of the soils change greatly from a certain depth. As shown from the table, the variation of pressuremeter parameters in three boreholes shows similar characteristics; however, when the depth is greater than 17 m, the COVs of shear modulus increases greatly. The statistical analysis in conjunction with the results of modulus degradation indicates that the heterogeneity of soil is increasing in the vertical direction, especially when the depth is more than 17 m. This can be attributed to the deposit history of soils in the Taihu area, as a lot of biological remains and detrital sediment are seen to be deposited in the sedimentation process of lacustrine clay, resulting in some interlayers in soils at different depths. This may be the main reason for the high variability of pressuremeter parameters in the vertical direction, which indeed shows the typical characteristics of the lacustrine clays, i.e., as the depth increases, more subcategories of each soil type are interlaced.

Meanwhile, it can also be found from the table that the shear strain has a quite distinct effect on the COV distribution, i.e., as the strain increases, the COV decreases. This also shows a consistent result as previously reported, where the shear modulus is strain dependent, and as the strain increases, due to the influence of factors such as plasticity, etc. the discrepancies of responses from each type of soil are reduced. Therefore, careful consideration of the loaded state on the structure may be worthwhile when choosing an appropriate shear modulus in design or numerical analysis.

**Figure 15:**
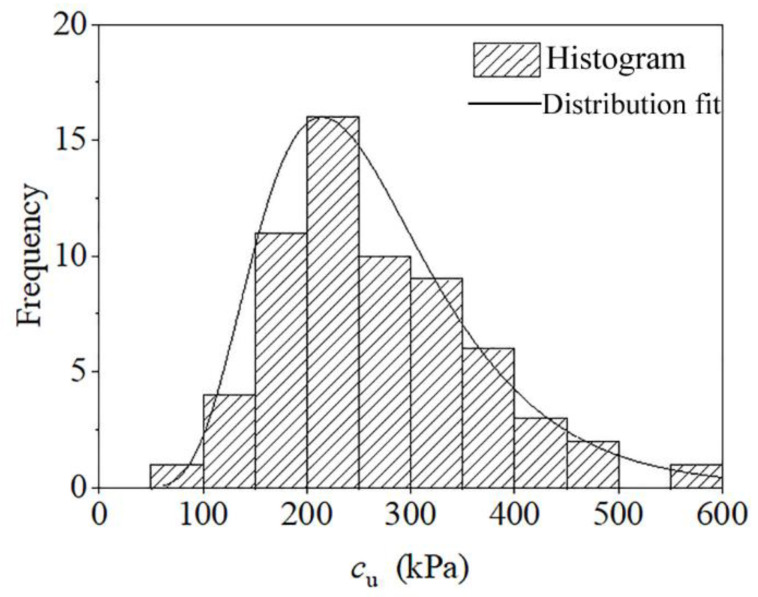
Histogram and the distribution fit of the undrained shear strength.

Moreover, from the test data, the distribution of the undrained shear strength and shear modulus can be estimated. Figure 15 shows that the undrained shear strength also follows a log-normal distribution. The estimation of the distributions is consistent with previous literature, such as Griffiths et al. [34,35] considered the undrained shear strength as log-normally distributed in a slope stability analysis, Jiang and Huang [36] developed a nonstationary random field model, and the undrained shear strength of clay is modeled as lognormally distributed.

**Figure 16:**
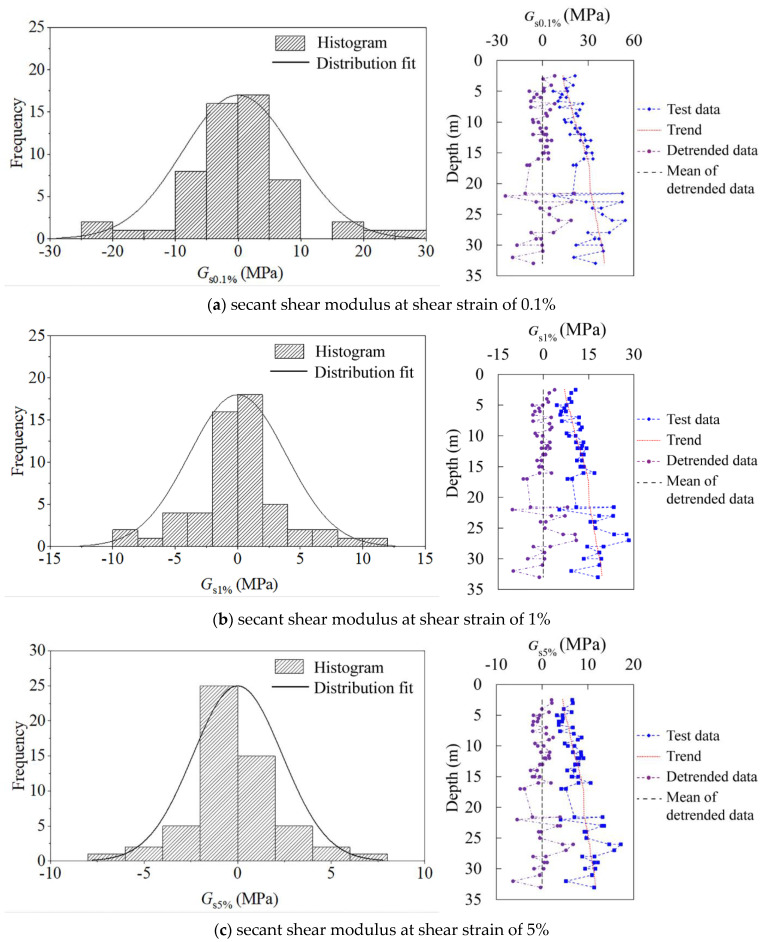

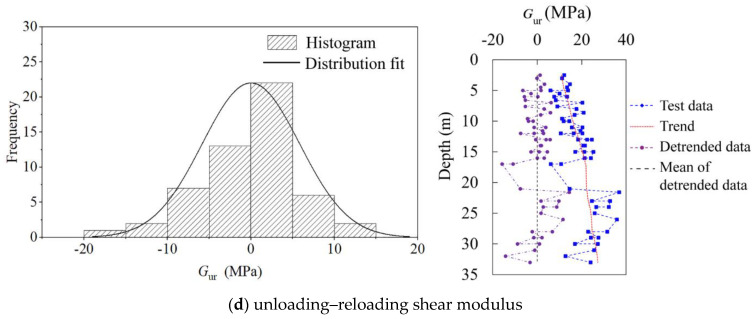
Histogram and the distribution fit of the detrended shear modulus.

Random field modeling of geotechnical spatial variability often decomposes the variation of geotechnical properties into the trend function (e.g., linear function in this study) and residue (so-called inherent spatial variability). Stationary random fields are then applied to model the residue. In other words, the detrending process is needed to enable the applicability of stationary random fields. Thereby, since there is a tendency of increased shear modulus with increased depth, the estimation of the distribution type has to be carried out under the situation of removing the linear trend. The detrending process is conducted by using MATLAB software. As shown in Figure 16, the right part presents the comparison between the original and detrended data of the secant shear modulus *G*_s_ from all three boreholes, the left part shows the detrended shear modulus and its histogram. It can be estimated from the histogram that the detrended *G*_s_ follows normal distributions. When the shear strain is 0.1%, the dominant distribution range of the detrended *G*_s_ is −10 to 10 MPa, while the shear strain increases to 5%, *G*_s_ mainly falls within the range of −2.5 to 2.5 MPa. This also shows a consistent result as previously reported, i.e., as the shear strain increases, the shear modulus is decreased (from an average value of 25 MPa at the shear strain of 0.1% to about 8 MPa when the shear strain increases to 5% for the original data). Similarly, the distribution of the detrended unloading–reloading shear modulus *G*_ur_ can also be estimated, as depicted in Figure 16d. The unloading–reloading shear modulus also follows a normal distribution, and the main distribution range varies from −10 to 10 MPa.

### 4.6. Scale of Fluctuation of the secant Shear Modulus and Undrained Shear Strength

In addition to the mean and standard deviation of soil parameters, the spatial correlation (i.e., the scale of fluctuation in this paper) has been recognized as an important parameter that can affect the probabilistic outcomes. The scale of fluctuation can be estimated from the in situ test results, based on the following equation [37]:

(6) $$\overset{\wedge }{\rho }(\tau )=\frac{\overset{\wedge }{\gamma }(\tau )}{\overset{\wedge }{\gamma }(0)}$$

where ρ(τ) is the correlation function, $\hat{\gamma }\left(\tau \right)$ is the covariance function, and can be expressed as

(7) $$\overset{\wedge }{\gamma }(\tau )=\frac{1}{N-1}\sum_{j=1}^{N}\left(x_{j}-\overset{\wedge }{\mu }\right)(x_{j+\tau }-\overset{\wedge }{\mu })$$

where *N* is the number of pairs of data at a lag distance of τ, $\overset{\wedge }{\mu }$ is the estimated mean of data, τ is the lag distance, and *j* refers to the index numbering from *j* = 0, 1, 2, … *N*. It is also noted that when τ=0, $\overset{\wedge }{\gamma }(0)$ is the standard deviation of data.

A commonly applied exponential correlation function is used here to model the correlation of soil parameters at different positions [38], i.e.,

(8) $$\rho (\tau )=\exp(-\frac{2}{\theta }\left|\tau \right|)$$

where θ is the scale of fluctuation to be estimated. The scale of fluctuation is calculated based on minimizing the error as follows:

(9) $$E=\sum_{j=1}^{N}{\left(\rho (\tau )-\overset{\wedge }{\rho }(\tau )\right)}^{2}$$

The value of θ that minimizes *E* can be calculated by finding the root of the following expression:

(10) $$\frac{\partial E}{\partial \theta }=-\sum_{j=1}^{N}\frac{2\tau }{\theta^{2}}\left(\overset{\wedge }{\rho }(\tau )-\rho (\tau )\right)\;\rho (\tau )$$

which can be expressed as

(11) $$\sum_{j=1}^{N}\tau \left(\overset{\wedge }{\rho }(\tau )-\rho (\tau )\right)\;\rho (\tau )=0$$

**Figure 17:**
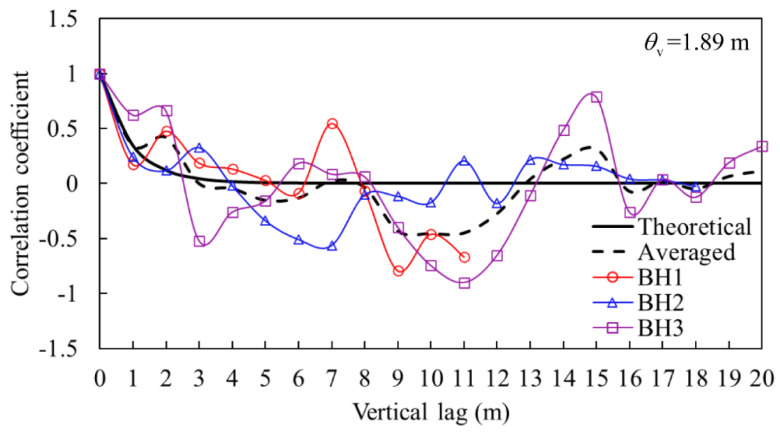
Vertical correlation length of the undrained shear strength.

In this paper, the vertical scales of fluctuation $\theta_{v}$ of the shear modulus and undrained shear strength are estimated based on the SBPM results. Figure 17 shows the theoretical and empirical correlation function of the undrained shear strength *c_u_*, which is the function of the relative distance between two different depths. The relative distance over depth in the correlation function is usually defined as the vertical lag [38]. The solid black line denotes the theoretical correlation estimated by using Equation (7), while the dashed black line indicates the average of three boreholes. The best-fit scale of fluctuation is determined by using Equation (10). From the data, the scale of fluctuation of the undrained shear strength is estimated to be 1.89 m.

**Figure 18:**
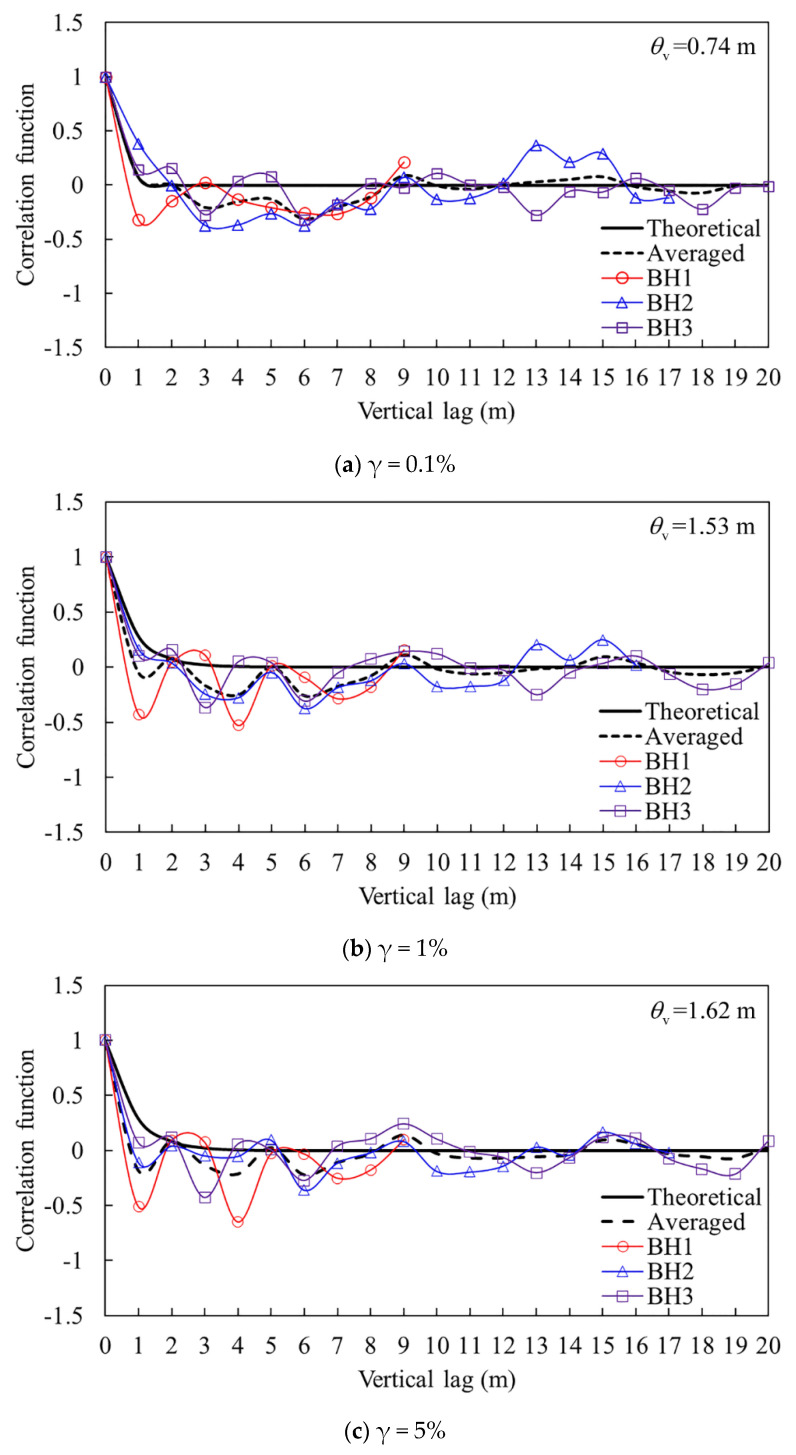
Vertical correlation lengths of the secant shear modulus at different shear strains.

Similarly, Figure 18 shows the calculated correlation lengths of the shear modulus *G*_s_ at different shear strains. It can be seen from the figure that the result of *G*_s__,0.1%_ suggests an average scale of fluctuation of 0.74 m ($\theta_{v}$ = 0.74 m), while the average of *G*_s__,1%_ and *G*_s__,5%_ are $\theta_{v}$ = 1.53 m and $\theta_{v}$ = 1.62 m, respectively. This more clearly suggests that as the loaded state is changed from a small-strain state (e.g., 0.1%) to a large one (e.g., 1%, 5%), the responses of the soil change greatly, with the correlation length being increased to be over two times. While the strain rises up from 1% to 5%, the correlation length hardly changes. This also can suggest a stable state of the soil being attained when the strain increases over 1% as mentioned above but with an indirect summary of the correlation lengths of the shear modulus at different shear strains. Meanwhile, compared to the scale of fluctuation of the undrained shear strength, it should be emphasized that the spatial correlation of the shear modulus along the vertical direction at small strains should be much less than that of the undrained shear strength.

## 5. Conclusions

A comprehensive interpretation of the SBPM test results on the lacustrine clay in Taihu Lake, China, is presented in this paper. Parameters, such as initial horizontal geo-stress, undrained shear strengths, and shear modulus, are analyzed. By comparing with the laboratory results of the undrained shear strength, as well as the analytical solutions for the initial pore water pressure distribution, a generally consistent pattern is shown, which demonstrates the accuracy of the SBPM test results.

As for the shear modulus, a generally increasing trend along the depth is provided, which is mainly attributed to the overburden stress. However, it should be noted that the strain is shown to have a great impact on the magnitude of the modulus, which can slow down its increasing rate, i.e., as the strain increases, the trend of the modulus increase is shown to gradually reduce. Strong degradation characteristics of the shear modulus are presented, where it can be seen that once the loaded strain increases, the shear modulus drops rapidly, and until the strain exceeds 1%, the modulus would come to be constant. The unloading–reloading shear modulus is shown to be mainly distributed within the strain range of 1–3.2% and thereby would be considered unsafe, especially for the foundation engineering where the strain change is normally very small.

A relationship between the secant shear modulus and the plasticity index is given in the paper, where it is found that the larger the plasticity index is, the higher the shear modulus would be, and meanwhile, the decay rate would also be higher. Statistical analyses of the pressuremeter parameters show that the undrained shear strength follows a log-normal distribution, while the shear modulus follows a normal distribution. Moreover, by summarizing the correlation lengths of the shear modulus at different shear strains, a distinct effect of the shear strain on the shear modulus change can also be observed, and the spatial correlation of the shear modulus along the vertical direction at small strains should be much less than that of the undrained shear strength.

## Figures and Tables

**Table 1:** Basic physical properties of samples obtained by laboratory tests.

Sample No.	Depth (m)	Specific Gravity	Water Contents (%)	Void Ratio	Density (g/cm^3^)	Liquid Limit (%)	Plastic Limit (%)	Plasticity Index
TH-SC1	3.0–3.5	2.72	27.3	0.835	1.92	35.1	20.6	14.5
TH-S	8.0–8.5	2.70	31.1	0.774	1.96	33.2	22.7	6.9
TH-SSC	13.0–13.5	2.74	36.4	1.099	1.91	38.4	19.0	15.4
TH-SC2	18.0–18.5	2.74	23.2	0.677	2.00	35.0	19.6	15.3
TH-SC2	21.0–21.5	2.72	22.0	0.746	2.02	32.9	16.5	16.4
TH-SC2	30.0–30.5	2.72	29.5	0.996	1.88	31.2	15.6	15.6
TH-C	41.0–41.5	2.74	26.6	0.709	1.94	36.9	21.4	15.5

Note: TH refers to Taihu lake, where the samples were obtained; SC, silty clay; S, silt; SSC, soft silty clay; C, clay.

**Table 2:** Statistics of the secant shear modulus and undrained shear strength.

Depth (m)	Statistics Parameters	*G*_s0.05%_ (MPa)	*G*_s0.1%_ (MPa)	*G*_s0.5%_ (MPa)	*G*_s1%_ (MPa)	*G*_s5%_ (MPa)	*C*_u_ (kPa)
≤17	Mean	26.17	21.03	12.77	10.33	6.36	264.91
	Standard deviation	9.38	6.92	3.57	2.77	1.71	91.23
	COV (%)	35.83	32.91	27.95	26.81	26.82	34.44
>17	Mean	45.89	36.90	22.33	18.02	11.00	280.30
	Standard deviation	17.80	13.62	7.39	5.72	3.28	79.94
	COV (%)	38.79	36.92	33.09	31.76	29.83	28.52

## Data Availability

All field test data and laboratory test results for this study are available from the corresponding author by request.
